# Exploring the Utility of NK Cells in COVID-19

**DOI:** 10.3390/biomedicines10051002

**Published:** 2022-04-26

**Authors:** Xuewen Deng, Hiroshi Terunuma, Mie Nieda

**Affiliations:** 1 Biotherapy Institute of Japan, Inc., 2-4-8 Edagawa, Koto-ku, Tokyo 135-0051, Japan; terunuma@bij-net.com (H.T.); nieda@bij-net.com (M.N.); 2 N2 Clinic Yotsuya, 5F 2-6 Samon-cho, Shinjuku-ku, Tokyo 160-0017, Japan

**Keywords:** SARS-CoV-2, COVID-19, NK cell, immune dysregulation, immunotherapy

## Abstract

Coronavirus disease 2019 (COVID-19) can manifest as acute respiratory distress syndrome and is associated with substantial morbidity and mortality. Extensive data now indicate that immune responses to SARS-CoV-2 infection determine the COVID-19 disease course. A wide range of immunomodulatory agents have been tested for the treatment of COVID-19. Natural killer (NK) cells play an important role in antiviral innate immunity, and anti-SARS-CoV-2 activity and antifibrotic activity are particularly critical for COVID-19 control. Notably, SARS-CoV-2 clearance rate, antibody response, and disease progression in COVID-19 correlate with NK cell status, and NK cell dysfunction is linked with increased SARS-CoV-2 susceptibility. Thus, NK cells function as the key element in the switch from effective to harmful immune responses in COVID-19. However, dysregulation of NK cells has been observed in COVID-19 patients, exhibiting depletion and dysfunction, which correlate with COVID-19 severity; this dysregulation perhaps contributes to disease progression. Given these findings, NK-cell-based therapies with anti-SARS-CoV-2 activity, antifibrotic activity, and strong safety profiles for cancers may encourage the rapid application of functional NK cells as a potential therapeutic strategy to eliminate SARS-CoV-2-infected cells at an early stage, facilitate immune–immune cell interactions, and favor inflammatory processes that prevent and/or reverse over-inflammation and inhibit fibrosis progression, thereby helping in the fight against COVID-19. However, our understanding of the role of NK cells in COVID-19 remains incomplete, and further research on the involvement of NK cells in the pathogenesis of COVID-19 is needed. The rationale of NK-cell-based therapies for COVID-19 has to be based on the timing of therapeutic interventions and disease severity, which may be determined by the balance between beneficial antiviral and potential detrimental pathologic actions. NK cells would be more effective early in SARS-CoV-2 infection and prevent the progression of COVID-19. Immunomodulation by NK cells towards regulatory functions could be useful as an adjunct therapy to prevent the progression of COVID-19.

## 1. Introduction

Coronavirus disease 2019 (COVID-19) caused by severe acute respiratory syndrome coronavirus 2 (SARS-CoV-2) emerged in late 2019 [1], and was declared a pandemic by the World Health Organization (WHO) [2]; it seriously devastated public health and economies worldwide. The spectrum of clinical presentations of COVID-19 individuals ranges from asymptomatic or mild upper respiratory symptoms to severe viral pneumonia causing acute respiratory failure [3,4]. Transmission of SARS-CoV-2 by asymptomatic individuals poses a great public health challenge in containment efforts [5]. The main characteristic symptoms of COVID-19 include fever, fatigue, dry cough, and respiratory distress [3,4]. Severe COVID-19 cases are characterized by pneumonia involving both lungs [6], which can progress to acute respiratory distress syndrome (ARDS), multiorgan failure, and even death [3,4,6,7].

Clinically, the disease symptoms vary depending on the prevalent strain, but most infected patients are asymptomatic. Those who develop pneumonia require hospitalization, a few of whom become severely ill [3]. Approximately 50% of patients with severe COVID-19 had a comorbidity, with hypertension being the most common, followed by diabetes, obesity, and coronary heart disease [3,6,8,9]. A study has shown increasing odds of death associated with older age [9]. Initially, the clinical management of COVID-19 consists of infection prevention and control measures, and supportive care such as oxygenation and mechanical ventilatory support. Current evidence-based treatment guidelines are regularly updated by the National Institutes of Health [10]. The direct-acting antivirals ritonavir-boosted nirmatrelvir (Paxlovid), remdesivir (Veklury), and molnupiravir, and anti-SARS-CoV-2 mAb products (either bamlanivimab plus etesevimab or sasirivimab plus imdevimab or sotrovimab) have recently been approved in the clinical management of COVID-19 patients [10].

Obviously, the pathogenicity of SARS-CoV-2 is largely related to the interplay between the virus and the host [11,12]. Indeed, the interaction between SARS-CoV-2 and host antiviral immunity, including innate and acquired immune responses, have come into the spotlight in this context as well [7,13,14,15]. Severe COVID-19 cases are associated with hypercytokinemia, or the cytokine storm, which is due to exaggerated immune responses leading to severe lung damage [7,15,16]. Analysis of bronchoalveolar immune cells has revealed the roles of macrophages and neutrophils in the pathogenesis of severe COVID-19 [17,18,19]. Autopsy studies of patients who died of COVID-19 provide new insights into the pathophysiology of severe COVID-19: lungs were highly inflamed and had dense infiltrations of aberrantly activated monocyte-derived macrophages and alveolar macrophages, but there was no significant increase in the count of T cells and natural killer (NK) cells in the lungs [20]. 

To date, it has remained elusive how SARS-CoV-2 infection disrupts the host immune homeostasis and stimulates a hyperactive pro-inflammatory response [13,15]. A proposed model has suggested that the ability of SARS-CoV-2 to evade innate immune responses plays a critical role [21]. SARS-CoV-2 can effectively evade the immune system, causing an inadequate or delayed response [22]. This immune escape gives rise to unrestrained SARS-CoV-2 replication, which eventually results in hyperactivated pro-inflammatory responses [21,23]. NK cells are critical in innate immune responses as essential frontline responders to viral infections [24]. SARS-CoV-2-specific NK cell responses have been observed in vaccinated macaques [25], as well as in patients convalescing from COVID-19 [26]. Human NK cells were found to have anti-SARS-CoV-2 activity [27,28] and antifibrotic activity [27]. Accordingly, NK cell receptors affect the clearance of SARS-CoV-2 [29] and COVID-19 progression [30,31,32,33,34,35]. In addition, a high NK cell count is associated with viral load decline [28], a short time duration of SARS-CoV-2 viral RNA shedding, a stronger antibody response, and a higher survival rate in COVID-19 patients [36,37]. Hence, increasing the count of functional NK cells might help in the management of COVID-19 through the elimination of SARS-CoV-2-infected cells at an early stage and inhibition of disease progression. Consequently, the use of NK cells has been proposed as a suitable therapeutic approach for COVID-19 patients [38]. However, our understanding of the role of NK cells in COVID-19 remains limited. In addition to their beneficial antiviral role, NK cells could also possibly pathologically damage host tissues in viral infections [39,40]. Thus, there is an urgent need to explore the utility of NK cells in SARS-CoV-2 infection by reviewing reported data obtained from COVID-19 patients to deepen our understanding of the role of NK cells in COVID-19, and to improve the therapeutic approaches for the prevention of SARS-CoV-2 infection and treatment of COVID-19 patients.

## 2. NK Cells

NK cells are innate immune cells that play essential roles in immunosurveillance against viral pathogens and cancer [41,42], while also regulating adaptive immune responses [43,44]. They work through a non-self-strategy in which they act against cells that do not express major histocompatibility complex class I (MHC-I) molecules, which mark cells as “self”. MHC-I-specific inhibitory receptors on NK cells include killer cell immunoglobulin-like receptors (KIRs) and lectin-like CD94–NKG2A heterodimers, which identify self-cells and prevent NK cells from being activated [45,46]. The activating receptors activated by pathogens and cellular stress induce signaling pathways, e.g., NKG2D, NKp30, and NKp46, which trigger NK cell responses [45,46]. NK cells kill target cells through various mechanisms: (i) direct lysis of target cells through cytotoxic degranulation by perforin and granzymes, or death receptors; (ii) production of inflammatory cytokines, such as interferon-γ (IFN-γ) and tumor necrosis factor-α (TNF-α), leading to indirect elimination of target cells; (iii) expression of CD16, which allows for the detection of antibody-coated target cells, thus leading to NK-cell-led antibody-dependent cell cytotoxicity (ADCC); and (iv) by interacting with other immune cells, such as monocytes, inducing IFN-γ production and enhancing cytotoxicity [47,48]. 

NK cells constitute 10–15% of the total lymphocytes in human peripheral blood [48,49]. NK cells are negative for CD3 and positive for CD16 or CD56, which are cell surface markers [48,49]. There are two major types of NK cell: CD56^dim^ CD16^bright/+^ (CD56^dim^) and CD56^bright^ CD16^dim/−^ (CD56^bright^) NK cells. The CD56^dim^ NK cells are the major subset, constituting 90% of NK cells in peripheral blood, and they have low cytokine production but have high cytotoxicity. They are involved in ADCC. The CD56^bright^ NK cells constitute the remaining 10%, and they produce abundant cytokines but have low natural cytotoxicity [49,50,51]. However, CD56^bright^ NK cells can also become cytotoxic upon appropriate activation, and play roles in different disease states, such as cancers and infections [52].

In the human lung, NK cells comprise up to 20% of all lymphocytes, and are composed of three different subsets: CD56^dim^, CD56^dim^CD16^−^, and CD56^bright^ NK cells [53,54]. CD56^dim^ NK cells are the vast majority (up to 80%) of lung NK cells, with the mature NK cells mainly circulating in the lung [55]. The remaining subsets correspond to either an intermediate stage of differentiation or recently activated NK cells that have lost CD16 expression on their surfaces [53]. CD49a^+^CD69^+^CD103^+^ NK cells reside specifically in the lung, representing <3% of the total lung NK cells, most of which are CD56^bright^ NK cells [53,54]. Lung CD56^dim^ NK cells are hypofunctional against target cells in terms of cytotoxicity, ADCC, and following stimulation by IFN-α [55]. By contrast, CD49a^+^CD56^bright^ lung-resident NK cells show a higher capacity to degranulate and to produce IFN-γ upon contact with virus-infected autologous macrophages in vitro, as compared with matched peripheral blood CD56^bright^ NK cells [54]. As a whole, in the lung, the predominant circulating NK cells are highly differentiated but hypofunctional, whereas lung-resident NK cells are hyperfunctional [53,54,55].

## 3. NK Cells in Viral Infections

NK cells play an important role in innate immune responses to viral infections [24,56,57,58]. The critical role of NK cells in eliminating viral pathogens is highlighted by a case report of a patient lacking NK cells who was highly susceptible to herpesvirus infection [59]. Subsequent studies of NK cell deficiency disorders, which are associated with the complete or partial impairment of NK cells in terms of their number and function, have been associated with an increased susceptibility to viral infections, as well as to more severe and progressive diseases [56]. These studies established the critical role of functional NK cells in the control of viral infections in humans [57,59]. 

Viral infections trigger a panel of common modifications in their respective target cells, which can be sensed by NK cells; NK cells utilize various recognition modes in viral infections [57]. The functional outcome of NK cells is determined by the integration of both activating and inhibitory signals that regulate NK cell activity [45,46,60]. Virus-infected cells show down-regulation in MHC-I expression [61,62], which decreases the frequency of inhibitory signals on NK cells [44,45,46]. Moreover, virus-infected cells expressing defined ligands can be directly targeted through activating receptors, such as natural cytotoxicity receptors (NKp30, NKp44, NKp46, and NKG2D [57,58,60]); e.g., NKp30 recognizes pp65 of human cytomegalovirus (CMV), NKp46 recognizes hemagglutinin of the influenza virus, and human NKG2D recognizes CMV glycoprotein UL16-binding proteins and the MHC-I-related molecules MICA and MICB [57,58,60]. Additionally, NK cells directly recognize viral moieties via the engagement of pathogen-associated molecular patterns [63] or transmembrane-activating receptors, such as human NKG2C [26,64]. Moreover, NK cells can be activated via CD16-mediated ADCC [48,65] or cytokines, such as interleukin (IL)-2, IL-12, IL-15, IL-18, and type I IFN, which can be produced by virus-infected cells and/or antigen-presenting cells [66]. As a result, NK cells are activated to kill virus-infected cells through the release of cytotoxic granules (containing perforin and granzymes) and death receptors, causing direct lysis and release of cytokines and chemokines, such as IFN-γ and TNF-α, to enhance cytotoxicity, while also regulating the adaptive immune responses [43,44]. These features suggest that NK cells serve as the major antiviral effector cells during the time that the acquired immune system is still being mustered [24,57] and virus-infected cells are developing mechanisms to escape T cell surveillance [62]. 

However, NK cells are also possibly associated with immunopathology in viral infections, such as influenza A virus (IAV) [39] and hepatitis B virus (HBV) [40] infections. NK cells are activated during viral infections [39,40]. The activated NK cells by IAV infection, which are responsive to the normally NK-cell-resistant lung epithelial cell line A549, could contribute to the killing of non-infected epithelial cells [39]; furthermore, the activation status of NK cells is related to the severity of e-antigen-negative HBV infection [40]. By contrast, in human immunodeficiency virus (HIV) and hepatitis C virus (HCV) infections, NK cells appear to act as a rheostat by eliminating activated CD4^+^ T cells that affect CD8^+^ T cell function and exhaustion, thus preventing T-cell-mediated pathology [43]. Additionally, NK cells crosstalk with myeloid cells, such as monocytes, macrophages, dendritic cells (DCs), and neutrophils in antiviral responses [67,68]. In particular, NK cells can effectively counteract virus-infected monocytes and macrophages, which produce large amounts of pro-inflammatory cytokines and chemokines, contributing to local inflammation and a dangerous systemic inflammatory response called the cytokine storm [69]. Therefore, when we examine the role of NK cells in SARS-CoV-2 infection, which is causing the ongoing COVID-19 pandemic, we should keep in mind the double-edged roles of NK cells in viral infections and inflammation-driven damage [70].

## 4. NK Cells in COVID-19 Patients

NK cells have become the focus of interest since the initial report of SARS-CoV-2 infection [71]. Data on NK cells in COVID-19 patients continue to rapidly accumulate [26,27,28,30,31,33,34,71,72,73,74,75,76,77,78,79,80,81,82,83,84,85,86,87,88,89,90]. Here, we give a detailed description of NK cells in COVID-19 patients.

### 4.1. Count and Frequency

In association with lymphopenia in COVID-19 patients, the NK cell count in peripheral blood is consistently and significantly decreased [27,71,72,73,74,75,76,77,78,79,80,81,82,83,84,85], and the count is inversely correlated with COVID-19 severity [27,71,73,74,75,77,78,80,81,82,83,84,85]. NK cell frequency is either decreased [74,87,88,90] or not significantly altered [33,34,75,77,78,79,84,85,89]. However, the NK cell count is restored to normal levels after clinical recovery from COVID-19 [71,78]. The depletion of NK cells in the peripheral blood in COVID-19 patients could potentially, in part, be due to their mobilization and homing to different tissue compartments. However, these cells may also, and more likely, be adversely affected by SARS-CoV-2 [27], whereby the virus-infected NK cells are activated, leading to their death during infection. These mechanisms may underlie the depletion of NK cells in COVID-19 patients [27,91,92]. However, these mechanisms need to be investigated further.

### 4.2. Subsets

NK cells are typically divided into subsets of cytokine-producing CD56^bright^ NK cells and cytotoxic CD56^dim^ NK cells [49]. Most studies have shown that the proportions of CD56^bright^ NK cells are decreased [27,72,77,79,86,87,88], with some exceptions [33,74,90]. However, findings on CD56^dim^ NK cells are conflicting: many results have shown decreased proportions [27,34,74,77,87,88], while some others have shown increased proportions [72,79] or no change [33,86,89,90] in COVID-19 patients. The reason for these conflicting results is currently unknown, and the correlation between NK cell subsets and patient conditions needs to be investigated in the future.

### 4.3. Immunotypes

NK cells play important roles in viral infections [24,56,57,58]; indeed, viral infections were found to correlate with the immunotypes of NK cells and disease progression [93]. Therefore, the immunotypes of NK cells have been intensively studied in COVID-19 patients [26,27,33,34,74,75,77,79,81,86,88,89,90]. NK cells become activated with increased expression levels of CD38 [27,75,90], HLA-DR [27,75,77,86], CD69 [27,77,79,86,90], and Ksp37 [77]. CD38 is a type II 45 kDa glycoprotein usually present on the surface membrane of NK cells, and physically/functionally clustered with FcγR III/CD16 [94]. HLA-DR-expressing NK cells are functionally activated [95]. CD69 is a triggering molecule [96] associated with early activation [97] and tissue residency [98]. Ksp37-expressing cells also express perforin, indicating their cytotoxic potential [99]. Additionally, NK cells exhibit a skewing to exhaustion due to increased expression levels of NKG2A [71,81,86,88], Tim-3 [75,77,79], CD244 [74], PD-1 [26,74,79], and CD39 [81], or increased proportions of “exhausted” CD56^dim^ CD16^−^ [33,34,74] and CD56^−^CD16^+^ [74,86] NK cells. The upregulated NKG2A expression in NK cells was found to be mediated by the viral spike protein [100]. However, SARS-CoV-2 encodes the HLA-E-stabilizing peptide Nsp13, which abrogates the inhibition of NKG2A-expressing NK cells [101]. Expansion of the CD56^dim^ CD16^−^ NK cell subset in COVID-19 patients was accompanied by decreased NK activity [33,34] and prolonged recovery of patients with severe COVID-19 [34]. The aforementioned exhausted NK cells have also been demonstrated in other viral infections, such as NKG2A^+^ and CD56^−^CD16^+^ NK cells in HBV infection [102,103]; Tim-3^+^, PD-1^+^, and CD39^+^ NK cells in HIV infection [104,105,106]; CD244^+^ NK cells in Epstein–Barr virus infection [107]; and CD56^dim^ CD16^−^ NK cells in HCV infection [108]. However, the increased expression levels of NKG2A [71], CD69, and Tim-3 [79] in NK cells are restored after clinical recovery from COVID-19. Moreover, the expression levels of NK-cell-activating receptors, such as NKG2D, sialic acid-binding Ig-like lectin 7 (Siglec-7), DNAX accessory molecule-1 (DNAM-1) [79,90], NKp46 [26], and CD16 [33,34,74], are decreased. The reduced expression levels of these activating receptors have been associated with reduced NK cell function in viral infections [109,110,111]. DNAM-1 expression in NK cells is critical to the clearance of SARS-CoV-2 and rapid recovery from infection [29]. In addition, the decreased CD16 expression level might be associated with abnormal NK cell maturation during infection or prolonged cellular activity/activation, driving CD16 shedding, which might be a regulatory mechanism to prevent activation-induced cell death [112]. Moreover, NK cells show increased expression levels of Ki-67 [77,88,90], CD98 [77], CXCR6, CD103 [86], the transcription factor Aiolos [79], caspase-3, and CD95 [27], which are related to cell cycling, metabolism, tissue residency, hematopoietic development, and apoptosis, whereas the expression level of the homing and extravasation marker CD49d is decreased [86]. Thus, the count of tissue-resident CD69^+^CD103^+^CXCR6^+^ NK cells increases in circulation, with a related surge of CD34^+^DNMA-1^bright^CXCR4^+^ inflammatory precursors from the bone marrow [86]. However, the count of CXCR6-expressing NK cells was decreased in another report [79]. Additionally, patients with poor outcomes have shown an expansion of CD56^dim^ CD57^+^ NKG2C^+^ [77,89] or CD56^+^CD57^+^FcεRIγ^−^ [79] adaptive NK cells. The expansion of adaptive NK cells is often associated with human CMV infection [113]. However, most of the patients are negative for CMV DNA in serum, and the expanded adaptive NK cells do not likely depend on CMV reactivation [77]. However, a later study has shown that the expansion of adaptive NK cells occurred mostly, but not exclusively, in individuals who were CMV-seropositive [89], raising the possibility of CMV reaction in COVID-19 patients. However, the expanded CD57^+^ NKG2C^+^ NK cells have been detected in convalescent COVID-19 patients with a SARS-CoV-2-specific response, raising the possibility that NK cell memory may also be generated [26]. In addition, perforin- and granzyme B-expressing CD56^bright^ NK cells have also been observed across COVID-19 disease states, which are driven by a defined inflammatory soluble factor, and correlate with disease severity [77,89]. Taken together, NK cells are robustly activated and exhausted in the peripheral blood of COVID-19 patients, and the counts of adaptive NK cells, armed CD56^bright^ NK cells, and bone-marrow-originating inflammatory precursors in circulation are increased in patients with severe COVID-19.

### 4.4. NK Cell Activity

NK cell activity in COVID-19 patients has been assessed on the basis of flow-cytometry-based CD107a (degranulation) expression. The percentage of CD107a-expressing NK cells is consistently lower in COVID-19 patients than in controls [27,28,71,72,79,86]. There is a statistically significant decrease in expression level of CD107a that is negatively correlated with C-reactive protein levels, indicating that, under conditions of exaggerated systemic inflammation, NK cells are dysfunctional in the peripheral blood of COVID-19 patients [79]. Incubation of NK cells with plasma from COVID-19 patients, especially plasma from patients with severe COVID-19, impaired CD107a expression in response to K562 cells compared with control plasma, suggesting the presence of soluble factors in the plasma that most likely contribute to the impairment [27]. TGF-β is one of these soluble factors that inhibit NK cell function [28].

### 4.5. Production of Cytokines and Chemokines

The antiviral effects of NK cells can be mediated through the cytokines they produce [24], in addition to crosstalks with other innate immune cells [67,68] and regulation of the adaptive immune response [43,44]. Therefore, the cytokine production ability of NK cells is an important parameter for NK cell function, in addition to NK cell activity. In the peripheral blood of COVID-19 patients, the ability of NK cells to produce cytokines, such as IL-2 [71], IFN-γ [27,71,79], and TNF-α [27,71,73], is reduced. Impaired IFN-γ and TNF-α production may be due to soluble factors in plasma from COVID-19 patients, as shown by a study on NK cell activity in response to K562 cell stimulation [27]. However, some studies do not report a significant decrease in IFN-γ production [72,73]. By contrast, the production of the chemokine MIP-1β (macrophage inflammatory protein-β) is increased [77]. These results indicate that the production of cytokines by NK cells in peripheral blood is impaired and exhibits a skewing towards the redistribution of NK cells to sites in the inflamed lung [77,92], which may be related to the decrease in the count of NK cells in circulation [27,71,72,73,74,75,76,77,78,79,80,81,82,83,84,85].

### 4.6. Functional Granule Components

The main mechanism by which NK cells eliminate virus-infected cells or transformed cells involves granule exocytosis, with direct release of cytolytic granules containing perforin and granzymes that kill target cells via apoptosis [114]. To explore the functional granule components in NK cells in COVID-19 patients, the expression levels of perforin, granzyme A (GrA), and granzyme B (GrB) in NK cells have been investigated. Data on functional granule components are inconclusive. The perforin expression level is increased [28,77,80,89,90], with some exceptions [73,86]. Regarding the expression levels of granzymes in COVID-19 patients, the results varied among studies: The expression level of GrA increased in [80] and decreased in [73], whereas that of GrB increased in [28,77,89] and decreased in [71,80]. The discrepancies among studies might be caused by the time of sampling during the disease course and disease severity.

### 4.7. Genetic Variants

NK cells rely on inhibitory and activating receptor signals to determine which target cells will be attacked or tolerated [45,46]. NKG2C is an activating NK cell receptor encoded by *KLRC2*; it binds to human leukocyte antigen (HLA)-E, leading to NK cell activation [30]. Heterozygous or homozygous *KLRC2* deletion may naturally occur, and is associated with a significantly low NKG2C expression level, or the absence of expression [30]. In addition, HLA-E* 0101/0103 genetic variants occur, which are attributable to a single-nucleotide polymorphism, and the cell surface expression level of HLA-E* 0101/0101 is lower than that of HLA-E* 0103/0103 [30]. The deletions of *KLRC2* and, at a lower degree, the HLA-E* 0101 allele are independent risk factors for severe COVID-19, suggesting that genetic variants in the NKG2C/HLA-E axis have a significant impact on COVID-19 severity. Detection of these variants my help identify patients at high risk of developing severe COVID-19 [30]. Moreover, KIRs expressed on NK cells are critical in regulating NK cell responsiveness through their binding to HLA class I ligands on cells [45,46]. A study has shown that the reduced gene coding for the activating receptor KIR2DS2 is associated with severe COVID-19 [31]. In particular, the frequency of the KIR2DS2/HLA C1 functional unit was reduced in severe COVID-19, suggesting a protective effect against adverse outcomes of COVID-19, which is probably achieved through the effective activity of NK cells, and thereby contributes to viral clearance in the early stages of SARS-CoV-2 infection [31]. In line with these studies, higher frequencies of the functional A-telomeric activating receptor KIR2DS4 [35] and the inhibitory receptors KIR2DL1 and KIR2DL1/S1 [33] are associated with severe COVID-19, whereas a higher frequency of activating receptor KIR3DS1 in the presence of HLA-B*15:01 is associated with mild and/or moderate COVID-19 [35].

## 5. Dysregulation of NK Cells in COVID-19 Patients

**Figure 1:**
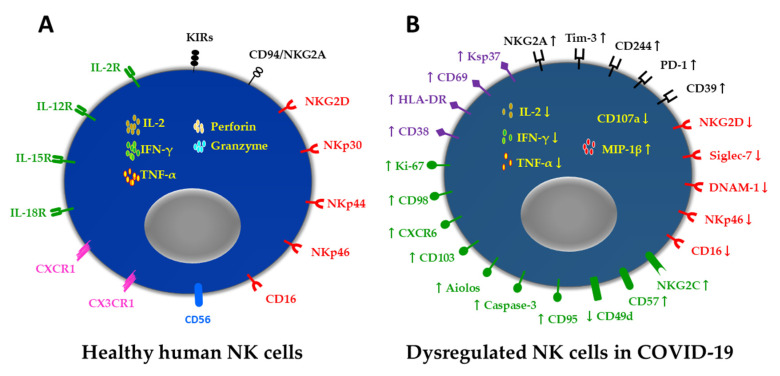
Schematic representation of human NK cells in healthy individual and COVID-19 patient. (**A**): Healthy human NK cells express the surface molecules CD56 and CD16; the inhibitory receptors MHC-I-specific killer cell immunoglobulin-like receptors (KIRs) and lectin-like CD94–NKG2A heterodimers; the activating receptors of NKG2D; natural cytotoxic receptors NKp30, NKp44, and NKp46; the cytokine receptors of IL-2, IL-12, IL-15, and IL-18; the chemokine receptors CXCR1 and CX3CR1; the effector molecules of cytokines IL-2, IFN-γ, and TNF-α; and cytolytic granules containing perforin and granzyme [45,46,49,52]. (**B**): In the peripheral blood of COVID-19 patients, dysregulation of NK cells has been observed, the cells exhibiting depletion and dysfunction. They show increased expression levels of CD38, HLA-DR, CD69, and Ksp37, and they exhibit a skewing to exhaustion, with increased expression levels of inhibitory receptors, such as NKG2A, Tim-3, CD244, PD-1, and CD39. Activating receptors with decreased expression levels include NKG2D, Siglec-7, DNAM-1, NKp46, and CD16. As a consequence, NK cells secrete reduced amounts of cytokines, such as IL-2, IFN-γ, and TNF-α, and show reduced degranulation, such as CD107a expression, whereas the production of chemokine MIP-β is increased. Moreover, NK cells show increased expression levels of Ki-67, CD98, CXCR6, CD103, Aiolos, caspase-3, and CD95, which are related to cell cycling, metabolism, tissue residency, hematopoietic development, and apoptosis, whereas the expression level of the homing and extravasation marker CD49d is decreased. In addition, the counts of NKG2C- and CD57-expressing adaptive NK cells are increased [26,27,28,33,34,71,72,73,74,75,76,77,78,79,80,81,82,83,84,85,86,87,88,89,90] (arrows indicate the surface receptors and intracellular molecules showing a change in expression level).

The immune responses to SARS-CoV-2 are key determinants of COVID-19 severity and outcome [11,12]; therefore, understanding the immunological underpinnings of COVID-19 pathogenesis is critical for the prevention of SARS-CoV-2 infection and COVID-19 treatment. NK cells are important in the immune defense against viral infections [24,56,57,58] and are the critical responders to SARS-CoV-2 infection [26,27,28]. However, dysregulation of NK cells has been observed, the cells exhibiting depletion and dysfunction in COVID-19 patients [26,27,28,33,34,71,72,73,74,75,76,77,78,79,80,81,82,83,84,85,86,87,88,89,90] (Figure 1), accompanied by expansions of adaptive NK cells [26,77,79,89] and more mature perforin- and granzyme B-armed CD56^bright^ NK cells [77,89], as well as the recruitment of CD34^+^DNAM-1^bright^CXCR4^+^ inflammatory precursors from the bone marrow [86].

**Figure 2:**
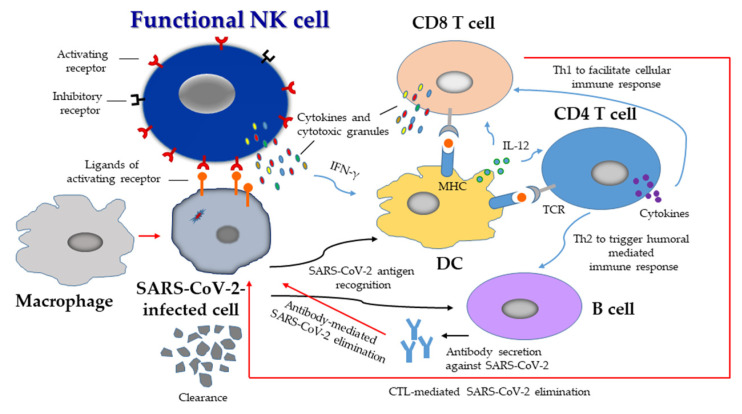
Schematic representation of immune responses to SARS-CoV-2 infection. Upon SARS-CoV-2 infection, local innate immune responses are initiated, that is, tissue-resident NK cells, dendritic cells (DCs), and alveolar macrophages respond rapidly in virus-infected tissues. NK cells can function as innate cytotoxic effectors as well as regulators modulating adaptive immunity to eliminate the virus. Functional NK cells exhibit balanced activation, that is, higher expression levels of activating receptors and lower expression levels of inhibitory receptors upon recognition of the virus in infected tissues. As a consequence, sufficient numbers of activated NK cells exert increased degranulation and cytokine secretion, initiate responses to eliminate SARS-CoV-2-infected cells in the early stage, and crosstalk with DCs to facilitate adaptive immune responses, as follows. DCs recognize the degraded SARS-CoV-2 antigen, activated NK cells secrete an array of cytokines, such as IFN-γ, that promote DC maturation with the establishment of a cytokine milieu, such as the release of IL-12, and DCs present the SARS-CoV-2-derived peptides to CD4 T cells and CD8 T cells through TCR–MHC interactions. Once exposed to the antigen, Th0 CD4 T cells polarize primarily towards Th1, leading to the release of cytokines, which facilitate antigen presentation to cytotoxic CD8 T cells (CTLs). CTLs detect SARS-CoV-2-infected cells and release cytotoxic granules, including granzyme B and perforin, to eliminate virus-infected cells. On the other hand, Th2 CD4 T cells facilitate antigen-recognized B cells to trigger humoral-mediated immune responses and antibody secretion involved in SARS-CoV-2 elimination.

The dysregulation of NK cells is associated with the severity of COVID-19. The depletion of NK cells from the peripheral blood may be due to the direct infection of NK cells by SARS-CoV-2 and/or the activation-induced cell death of NK cells during infection [27,91,115], as well as the mobilization/homing of NK cells to affected tissues [77,91,92]. NK cell function may be impaired by SARS-CoV-2 infection through, for example, the upregulation of inhibitory receptor NKG2A expression by the viral spike protein [100]. Additionally, an impaired IFN type I response of NK cells has been demonstrated in SARS-CoV-2 infection [15,27,75], which could be induced by type I IFN deficiency [75], prolonged and excessive IFN-α production [27], and autoantibodies against type I IFNs [116]. The type I IFN response in NK cells is crucial to antiviral defense [15,117], in conjunction with the ability to contain SARS-CoV-2 infection [118]. Moreover, hyperinflammation responses, such as elevated IL-6 levels [27,73,75,78,83,84,89] and an untimely early production of TGF-β [28] or other unknown soluble plasma components [27], might also result in the impairment of NK cells [27,28,119]. Furthermore, analysis of transcriptomic datasets of host responses to viral infections has revealed that the IL-15/IL-15RA axis plays a role in immune dysregulation [120], in which prolonged exposure of NK cells to high circulating IL-15 levels in a COVID-19 patient triggers NK cell dysfunction [121]. Effective immune responses to viral infections mostly depend on the adequate orchestration of both innate and adaptive immune responses to the virus. NK cells, DCs, macrophages, and granulocytes are innate immune cells, whereas T and B cells are adaptive immune cells. Presently, not much is known about the features of immune responses to SARS-CoV-2 that protect against severe COVID-19 because most cohorts profiled to date have included only hospitalized patients. Neutralizing antibodies and SARS-CoV-2-specific T cell responses have been detected in mildly symptomatic patients, providing evidence of an effective immune response across the disease spectrum [122]. Notably, patients with mild COVID-19 have much lower levels of pro-inflammatory plasma cytokines [18,84], suggesting that the immune response in mild COVID-19 can eradicate the virus without triggering the hyperinflammatory state observed in severe cases. NK cells are the first responders to viral infection [24,57]. Upon SARS-CoV-2 infection, local innate immune cells, such as tissue-resident NK cells, DCs, and alveolar macrophages, respond rapidly in SARS-CoV-2-infected tissues to initiate immune responses to eliminate the virus [44,67,68]. Functional NK cells exhibit balanced activation, in which the expression levels of activating receptors are high, whereas those of inhibitory receptors are low, upon recognition of SARS-CoV-2-infected cells [42]. As a consequence, a sufficient count of activated functional NK cells exert enhanced degranulation and cytokine secretion, and initiate innate cytotoxic function, to eliminate SARS-CoV-2-infected cells at an early stage [24,57]. Moreover, crosstalks of NK cells with DCs are facilitated to modulate adaptive immune responses [44,67,68]. DCs recognize the degraded SARS-CoV-2 antigen, and activated NK cells secrete an array of cytokines, such as IFN-γ, that promote DC maturation with the establishment of a cytokine milieu, such as the release of IL-12, and present the SARS-CoV-2-derived peptides to CD4^+^ T cells and CD8^+^ T cells through TCR–MHC interactions. Once exposed to the antigen, Th0 CD4^+^ T cells polarize primarily towards Th1, leading to the release of cytokines, which facilitate DC antigen presentation to cytotoxic CD8^+^ T cells (CTLs). CTLs detect SARS-CoV-2-infected cells and release cytotoxic granules, including GrB and perforin, to eliminate SARS-CoV-2-infected cells. On the other hand, Th2 CD4 T cells facilitate antigen-recognized B cells to trigger humoral-mediated immune responses and the secretion of antibodies involved in SARS-CoV-2 elimination [123,124,125] (Figure 2). A scenario of effective host anti-SARS-CoV-2 immunity emerges in asymptomatic infection and in mild upper respiratory symptomatic COVID-19 infection.

**Figure 3:**
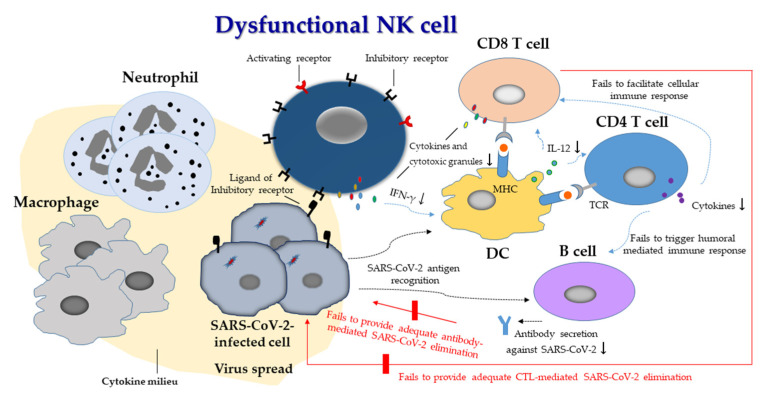
Schematic representation of dysregulated immune responses in COVID-19. NK cells are dysregulated in COVID-19, exhibiting depletion and dysfunction. The dysfunctional NK cells exhibit lower expression levels of activating receptors and higher expression levels of inhibitory receptors. As a consequence, NK cells show reduced degranulation and cytokine secretion. They fail to directly eliminate SARS-CoV-2-infected cells, and to indirectly, because of their impaired crosstalk with DCs, modulate adaptive immune responses. They fail to provide adequate CTL- and antibody-mediated SARS-CoV-2 elimination, allowing the virus to persistently replicate and spread, leading to further recruitment of infiltrating monocytes and granulocytes with the intent to initiate viral clearance. Together, these failures result in excessive and prolonged stimulation of the immune system, with the progressive accumulation of virus-infected cells and inflammatory myeloid cells to the sites of viral infections [17,18,19,20]; this in turn leads to the production of chemokines and cytokines and further recruitment of inflammatory cells, resulting in the dysregulated release of pro-inflammatory cytokines at elevated levels, contributing to over-inflammation and tissue injury [15,16,125]. (Arrows indicate the reduced degranulation and secretion of cytokines and antibodies).

Lessons learned from genetic syndromes of NK cell cytotoxic defects have implicated NK cells in the quality control of innate immune responses by inhibiting excessive myeloid responses, thereby preventing immunopathology [126,127]. The dysregulation of NK cells in COVID-19 patients results in not only the failure to eliminate SARS-CoV-2-infected cells directly [27,28] but also the indirect impairment of the crosstalks of NK cells with DCs to modulate adaptive immune responses [67,68]. Consequently, failure to provide adequate CTL- and antibody-mediated SARS-CoV-2 elimination allows the virus to persistently replicate and spread, leading to further recruitment of monocytes and granulocytes. As a result, the excessive and prolonged stimulation of the immune system, with the progressive accumulation of SARS-CoV-2-infected cells and inflammatory myeloid cells in sites of viral infection, occurs [17,18,19,20]; this in turn leads to the production of cytokines and chemokines [69], and to the further recruitment of inflammatory myeloid cells. These processes refer to the dysregulated release of pro-inflammatory cytokines at elevated levels, contributing to over-inflammation and tissue injury [15,16,128] (Figure 3). A scenario of dysregulated and exaggerated immune responses emerges in severe and critical COVID-19 patients.

Indeed, the lungs of severe COVID-19 patients are highly infiltrated with aberrantly activated macrophages and neutrophils, without significant increases in the counts of T cells and NK cells [17,18,19,20]. By contrast, more NK cells and T cells are accumulated, with fewer neutrophils and macrophages, in mild COVID-19 patients [129]; this suggests that NK cells do not participate in the exaggerated inflammatory responses observed in ARDS, but may facilitate immune–immune cell interactions to prevent over-inflammation and tissue injury [129]. Thus, NK cells function as key elements in the switch from an effective to a harmful immune response in COVID-19. Therefore, an adequate count of functional NK cells may strengthen the immunosurveillance against SARS-CoV-2 infection [42]. Indeed, dysfunction of NK cells is linked with increased SARS-CoV-2 susceptibility. Genetic variants [30,31], aging, obesity, unhealthy lifestyle [42], and cancer [130] cause NK cell dysfunction, which is associated with increased risk of severe COVID-19 [9,30,31,131,132,133]. Conversely, a higher count of NK cells is associated with a faster decline in viral load [28], a shorter time duration of SARS-CoV-2 viral RNA shedding, a better antibody response, and a higher survival rate in COVID-19 patients [36,37]. In addition, higher expression levels of the activating receptor DNAM1 on NK cells correlate with the rapid clearance of SARS-CoV-2 and recovery from COVID-19 [29].

Since late November 2021, the Omicron variant has emerged as the primary cause of COVID-19, and caused a huge increase in the reported incidences around the world [134]. Immune responses may vary between strains and variants of SARS-CoV-2 [134]. However, for this paper, data were collected before the emergence of the Omicron variant. Therefore, NK cell responses to the Omicron variant, and their differences between strains and variants, are currently not known owing to absence of genomic information. Moreover, NK cell responses to the currently prevalent Delta and Omicron variants may differ. The Omicron variant may initiate more effective immune responses, including NK cell responses, than other variants, especially the Delta variant; as such, variant-dependent responses should to be explored further.

## 6. NK-Cell-Based Therapies for COVID-19

Currently, the vaccines and drugs for SARS-CoV-2 have worked in improving the pandemic situation and outcome of COVID-19. We remain cautious, however, because mutant SARS-CoV-2 strains and new viruses will test our pandemic preparedness, requiring accelerated and swift deployment of experimental therapeutics to counter threats to human health. NK cells are important effectors in not only combating SARS-CoV-2 infection directly [27,28,101], but also indirectly by modulating adaptive immune responses to curtail infection through crosstalks with DCs [44,67,68] (Figure 2), and by inhibiting fibrosis progression [27]. Dysregulation of NK cells has been observed in COVID-19 patients, the cells exhibiting depletion and dysfunction [26,27,28,33,34,71,72,73,74,75,76,77,78,79,80,81,82,83,84,85,86,87,88,89,90]; NK cell dysfunction is linked with increased SARS-CoV-2 susceptibility [9,30,31,131,132,133], suggesting that dysregulation of NK cells may contribute to the worsening of COVID-19 (Figure 3). Therefore, therapies based on functional NK cells may provide potential therapeutic strategies to fight this COVID-19 pandemic. Restoration of the function of NK cells in patients with pathogenic germline mutation(s) in the X-linked IL-2 receptor subunit gamma gene via allogeneic hematopoietic cell transplantation can lead to persistent remission of human-papillomavirus-related diseases [135]. In addition, the function of NK cells is associated with lifestyle [42]. Therefore, adherence to a healthy lifestyle, such as adequate sleep, moderate exercise, forest bathing, and listening to music, and the avoidance of cigarette smoking, alcohol consumption, stress, and obesity, will be favorable for the immunosurveillance of SARS-CoV-2 infection [42].

Additionally, NK-cell-based cancer immunotherapies are well established [136,137]. Sources of therapeutic NK cells are currently being tested clinically, such as autologous NK cells, haploidentical NK cells, umbilical cord blood NK cells, cytokine-induced memory-like NK cells, and chimeric antigen receptor (CAR)-NK cells. Methods designed to augment the cytotoxicity and longevity of NK cells are also under clinical investigation, including cytokine-based agents, NK-cell-engager molecules, and immune-checkpoint inhibitors [137]. Moreover, CAR-NK cells that target SARS-CoV-2 have been developed, and they show superior killing activity and cytokine production [138,139].

Clinical trials of NK cell therapy in COVID-19 patients have begun (Table 1) [36,38,140]. The early results of a Phase I/II clinical trial of NK cell (CYNK-001) therapy for COVID-19 patients (NCT04365101) have been reported recently [141]. CYNK-001 is a cryopreserved, allogeneic, off-the-shelf NK cell product derived from placental CD34^+^ cells. In the Phase I trial focusing on the safety of administration, patients will receive up to three CYNK-001 infusions. Four of the six patients treated to date have been evaluated at the time of reporting. In all four patients, the three infusions were well tolerated. In three of the four patients, oxygenation improved after the first infusion of CYNK-001 and radiographic improvement was noted. The remaining patient developed progressive hypoxemia prior to the administration of the first infusion of CYNK-001, requiring a larger amount of supplemental oxygen. The patient received three infusions of CYNK-001, but the amount of oxygen required increased. Twelve days after the first CYNK-001 infusion, the patient died of respiratory failure, and it was not possible to rule out the contribution of CYNK-001. The other three patients were discharged with an average follow-up of 16 days after the first infusion [141]. However, more data from clinical trials are required.

Although studies have shown compelling evidence of a less severe disease and lower hospitalization rates associated with Omicron variant infection [142], the high transmissibility of this variant might still impact healthcare system readiness and increase the absolute number of hospitalizations. Nonetheless, the response of NK cells to the Omicron variant is yet to be explored. However, NK-cell-based therapies could contribute to the rapid control and clearance of viral infection via the preserved ADCC mediated by NK cells after vaccination or prior infection [143], thereby attenuating disease severity.

In addition, vaccines and antiviral drugs are invaluable in attenuating infection and accelerating SARS-CoV-2 clearance, as well as lowering mortality caused by the Delta variant [144]. However, individuals with immunocompromised conditions (e.g., organ transplantation and immunodeficient patients) may not be able to mount an effective immune response. NK-cell-based therapies, especially CAR-NK cell therapy [138,139], may provide an alternative strategy for patients unresponsive to current protection and/or treatment strategies, in preparation for future pandemics.

Furthermore, the dysregulation of NK cells upon COVID-19 infection may be due to impaired type I IFN responses, such as type I interferon deficiency [75], or elevated levels of proinflammatory cytokines, such as IL-6 [27,73,75,78,83,84,89] and/or TGF-β [28]. Therefore, the identification of possible populations that respond to immunomodulators [145], such as treatment with INF-α or INF-β, or Tocilizumab, in combination with NK-cell-based therapies, is attractive. Nonetheless, this is yet to be explored.

The rationale of NK-cell-based therapies for COVID-19 has to be based on the timing of therapeutic interventions and disease severity, which is determined by the balance between beneficial antiviral and potential detrimental pathologic actions. NK-cell-based therapies would be more effective early in the infection in order to initiate effective immune responses to prevent the progression of the disease (Figure 2). Immunomodulation of NK cells towards regulatory function by crosstalk with myeloid cells, especially to counteract virus-infected monocytes and macrophages (which produce large amounts of pro-inflammatory cytokines and chemokines, contributing to local inflammation and a dangerous systemic inflammatory response [69]), could be useful as an adjunct therapy for progressive COVID-19, thereby attenuating disease severity.

## 7. Future Directions and Concluding Remarks

The rapid spread of SARS-CoV-2 and the unprecedented nature of COVID-19 has required an urgent response worldwide [2]. Prevention of infection, vaccines, and antiviral drugs are important for ending this COVID-19 pandemic. Extensive data accumulated thus far have underlined the fact that immune responses to SARS-CoV-2 infection play a deterministic role in shaping the clinical course of COVID-19 [14,15,16,17,18,19,20]. NK cells are important in the control of viral infections [24,56,57,58], including SARS-CoV-2 infection [27,28,101]. Notably, the SARS-CoV-2 clearance rate, antibody responses, and COVID-19 progression correlate with NK cell status [28,29,36,37], and NK cell dysfunction is linked with increased SARS-CoV-2 susceptibility [9,30,31,131,132,133]. Thus, NK cells function as a key element in the switch from effective to harmful immune responses in COVID-19. However, dysregulation of NK cells correlates with COVID-19 severity [26,27,28,33,34,71,72,73,74,75,76,77,78,79,80,81,82,83,84,85,86,87,88,89,90], which perhaps causes disease progression. Hence, NK-cell-based therapies with sufficient antiviral capacities and a strong safety profiles in oncology may encourage the rapid application of these cells towards improving the management of COVID-19 by suppressing the virus, favoring finely tuned immune responses, and inhibiting fibrosis progression [27,28,38,101] (Table 1). Importantly, our knowledge of the role of NK cells in COVID-19 remains insufficient, such as the differences in their responses to different strains and variants, especially the current Delta and Omicron variants. Moreover, NK-cell-based therapies may potentiate the immunopathology of COVID-19 [70]. Thus, clear proof of protection, alongside increased knowledge regarding the pathology of NK cells in COVID-19, and differences in NK cell responses to different SARS-CoV-2 variants, is required. To this end, it is necessary to comprehensively study SARS-CoV-2-infected individuals with mild symptoms because they have effective immune responses, in addition to patients with severe COVID-19. It is important to study the early stages of SARS-CoV-2 infection to determine the interactions between infected cells and adjacent innate immune cells at primary sites of SARS-CoV-2 infection, lungs, and other tissues, since most studies of COVID-19 patients have been focused on peripheral blood. Additionally, models should help elucidate the role of NK cells in SARS-CoV-2 infection [146,147,148]. Such knowledge will guide us in how to modulate immune responses for maximum benefit and to improve the outcome of COVID-19 patients.

## Figures and Tables

**Table 1:** Ongoing clinical trials of NK cell therapy for COVID-19.

No.	NK Cell	Subject	Phase	Trial Identifier	Status	Location
1	Undisclosed	Not clear	I	NCT04280224	Recruiting	China
2	Allogeneic NKG2D-ACE2 CAR-NK cells enriched from umbilical cord blood and engineered genetically	Onset of illness <14 days	I/II	NCT04324996	Recruiting	China
3	Induced pluripotent stem cell (iPSC)-derived NK cells	Hospitalized patients	I	NCT04363346	Completed	Minnesota, USA
4	Human placental CD34+-derived and culture-expanded NK cells	Moderate disease	I/II	NCT04365101	Active, not recruiting	New Jersey, USA
5	Allogeneic NK cells from donors who have recovered from COVID-19	Onset of symptoms <10 days	I/II	NCT04578210	Recruiting	Spain
6	Undisclosed	Undisclosed	I	NCT04634370	Not yet recruiting	Brazil
7	Allogeneic NK cells derived from CD34+ hematopoietic stem cells	Hospitalized patients	I	NCT04900454	Recruiting	Seattle, USA

## Data Availability

Data sharing not applicable.
